# Predictors and Outcomes of Healthcare-Associated Infections Caused by Carbapenem-Nonsusceptible Enterobacterales: A Parallel Matched Case-Control Study

**DOI:** 10.3389/fcimb.2022.719421

**Published:** 2022-02-24

**Authors:** Grace S. R. Hoo, Yiying Cai, Yan Ching Quek, Jocelyn Q. Teo, Saugata Choudhury, Tse Hsien Koh, Tze Peng Lim, Kalisvar Marimuthu, Oon Tek Ng, Andrea L. Kwa

**Affiliations:** ^1^ Department of Pharmacy, Tan Tock Seng Hospital, Singapore, Singapore; ^2^ Department of Pharmacy, Singapore General Hospital, Singapore, Singapore; ^3^ Department of Pharmacy, National University of Singapore, Singapore, Singapore; ^4^ Programme in Health Services & Systems Research, Duke-National University of Singapore (NUS) Medical School, Singapore, Singapore; ^5^ Saw Swee Hock School of Public Health, National University of Singapore, Singapore, Singapore; ^6^ Department of Laboratory Medicine, Tan Tock Seng Hospital, Singapore, Singapore; ^7^ Dorevitch Pathology, Melbourne, VIC, Australia; ^8^ Department of Microbiology, Singapore General Hospital, Singapore, Singapore; ^9^ Singhealth Duke-National University of Singapore (NUS) Pathology Academic Clinical Programme, Singapore, Singapore; ^10^ Singhealth Duke-National University of Singapore (NUS) Medicine Academic Clinical Programme, Singapore, Singapore; ^11^ Department of Infectious Diseases, Tan Tock Seng Hospital, Singapore, Singapore; ^12^ Yong Loo Lin School of Medicine, National University of Singapore, Singapore, Singapore; ^13^ Hospital-Acquired Infection (HAI) Surveillance Unit, National Centre for Infectious Diseases, Singapore, Singapore; ^14^ Department of Infectious Diseases, National Centre for Infectious Diseases, Singapore, Singapore; ^15^ Lee Kong Chian School of Medicine, Nanyang Technological University, Singapore, Singapore; ^16^ Emerging Infectious Diseases, Duke-National University of Singapore, Singapore, Singapore

**Keywords:** carbapenem resistance enterobacteriaceae, healthcare-associated infections, risk factor analysis, predictors and outcome, case-control and matched study

## Abstract

**Objectives:**

The increasing incidence of carbapenem-nonsusceptible Enterobacterales as major pathogens in healthcare associated infections (HAIs) is of paramount concern. To implement effective prevention strategies against carbapenem-nonsusceptible Enterobacterales (CnSE) HAIs, it is crucial to identify modifiable factors associated with these infections. We identified risk factors for CnSE-HAIs, and compared clinical outcomes of CnSE-HAI and carbapenem-sensitive Enterobacterales (CSE)-HAI patients.

**Methods:**

We conducted a multi-centre parallel matched case-control study in two 1700-bedded Singapore acute-care hospitals from 2014–2016. Patients with CnSE-HAIs and CSE-HAIs were compared to a common control group without HAIs (1:1:3 ratio), matched by time-at-risk and patient ward. Carbapenem nonsusceptible was defined as non-susceptibility to either meropenem or imipenem. Presence of healthcare associated infections were defined by the criteria provided by the European Centre for Disease Prevention and Control. Outcomes of CnSE-HAI and CSE-HAI patients were compared using multivariable logistic and cox regression; the models were adjusted for infection and treatment characteristics.

**Results:**

Eighty CnSE-HAI and 80 CSE-HAI patients were matched to 240 patients without HAIs. All CRE-HAIs patients had prior antibiotic exposure, with 44 (55.0%) with prior carbapenem exposure. The most common CnSE-HAIs were intra-abdominal infections (28.8%) and pneumonia (23.8%). The most common CnSE species was *Klebsiella* spp. (63.8%). In the risk factor analysis, presence of drainage devices [adjusted odds ratio (aOR), 2.19; 95% CI, 1.29 – 3.70] and prior carbapenem exposure (aOR,17.09; 95% CI, 3.06 – 95.43) independently predicted CnSE-HAIs. In the crude outcomes analysis, CnSE-HAI patients had higher all-cause in-hospital mortality and longer time to discharge compared to CSE-HAI patients. After adjusting for differences in receipt of antibiotics with reported susceptibility to the Enterobacterales, there was no significant difference in all-cause in-hospital mortality between the two groups (aOR, 1.76; 95% CI, 0.86–3.58). Time to discharge remained significantly longer in patients with CnSE-HAI (adjusted hazard ratio, 0.71; 95% CI, 0.51 – 0.98) after adjusting for disease severity, receipt of antibiotics with reported susceptibility and receipt of appropriate source control.

**Conclusion:**

Appropriate management of deep-seated Enterobacterales infections and reducing exposure to carbapenems may reduce risk of CnSE-HAIs in Singapore. Efforts to improve antimicrobial therapy in CnSE-HAI patients may improve patient outcomes.

## Introduction

Modern medical care has evolved with increasingly invasive procedures and complex patients. This has contributed to the risk of healthcare-associated infections (HAIs) (World Health Organization, 2002). HAIs are currently one of the most frequent adverse events encountered by patients while receiving care in institutions worldwide. HAIs caused by Enterobacterales are a particularly important healthcare problem in Singapore (Gupta et al., 2011; Teo et al., 2016; Cai et al., 2017). In a 2015 Singapore point prevalence survey, it was found that the prevalence of HAI in acute-care hospitals was 11.9%; among the pathogens implicated, almost one-third were Enterobacterales (Cai et al., 2017).

Against severe HAIs caused by Enterobacterales, carbapenems are a class of broad-spectrum antibiotics typically used as last-resort option (Nordmann et al., 2012). However, the emergence of carbapenem-resistant Enterobacterales (CRE) as major nosocomial pathogens have compromised the utility of carbapenems (Nordmann et al., 2012). In Singapore hospitals, the incidence of CRE infections has been on an upward trend since 2012 (Koh et al., 2013; Teo et al., 2016). This rising trend, combined with the shrinking antimicrobial developmental pipeline, greatly limits effective treatment options and makes the combat against CRE-HAIs of paramount concern.

It has been estimated that approximately 20% to 70% of all HAIs are preventable (Scotts, 2009; Peleg and Hooper, 2010). To establish appropriate interventions targeted at reducing the incidence of CRE-HAIs, it is pertinent to recognise the associated risk factors. In Singapore, there have been several risk factor studies for CRE acquisition (Teo et al., 2012; Ling et al., 2015). Interestingly, none of these studies differentiated the predictors for colonisation and infection (Teo et al., 2012; Ling et al., 2015). In addition, none of the local studies differentiated the risk factors between nosocomial and community-acquired CRE infections (Teo et al., 2012; Ling et al., 2015). Hence, important risk factors that are specific for each patient type/setting may have been obscured. Our study aims to identify risk factors specifically associated with the development of carbapenem-nonsusceptible Enterobacterales (CnSE)-HAIs in Singapore. We also compare the clinical outcomes between patients with CnSE-HAIs and carbapenem-susceptible Enterobacterales (CSE)-HAIs.

## Materials and Methods

### Study Design and Setting

A multicentre retrospective matched case-case-control study was conducted from 2014 – 2016 at a 1,700-bed tertiary and a 1,700-bed secondary acute-care public hospital in Singapore. The study was conducted in accordance to the methods for identifying risk factors for antimicrobial-resistant pathogens, detailed by Kaye Kaye et al. (2005) Both hospitals have well-established multi-disciplinary antibiotic stewardship programmes (ASPs) that employ a multi-pronged audit-feedback approach (concurrent audit and feedback; guideline implementation) to promote appropriate carbapenem prescribing. In the secondary acute-care hospital, a clinical decision support system has been implemented to guide carbapenem prescribing during the study period (Teo et al., 2012; Lew et al., 2015). Both hospitals routinely performs CRE rectal swab or faeces surveillance in high-risk patients (e.g. ICU patients, patients transferred from an overseas hospital) (Ministry of Health Singapore, 2017). Infection control measures for CRE and HAIs remained largely unchanged in the study period. There was also no documented HAI or CRE outbreaks and no changes in management algorithm for HAIs and CREs in either institution during the study period. The study was reviewed and approved by both SingHealth and National Healthcare Group ethics review board (CIRB/2016/2388). As this was a retrospective study, informed consent was waived.

### Patient Population

All cases and controls were identified from institutional electronic databases. The first case group consisted of adult inpatients (≥21 years old) who acquired a HAI attributed to one or more microbiologically-confirmed CnSE. Only the first CnSE-HAI episode in the study period was included. The second case group consisted of adult inpatients (≥21 years old) who acquired a HAI attributed to one or more microbiologically-confirmed CSE. Patients who have both CSE- and CnSE-HAIs within a single admission were excluded from the second case group as inclusion of these patients in the second case group would have led to an overlap with patients in the first case group. Both CnSE-HAIs and CSE-HAIs were defined according to the European Centre for Disease Prevention and Control (ECDC) definition for HAIs; patients who did not meet the criteria for an active HAI as per ECDC were excluded from the study as they were deemed to be either community-acquired infection or colonised (European Centre for Disease Prevention and Control, 2013). The following Enterobacterales – *E. coli*, *Enterobacter* spp., and *Klebsiella* spp. were included as they were the most common Enterobacterales species associated with HAIs in Singapore (Cai et al., 2017). Carbapenem-non-susceptible was defined as non-susceptibility (intermediate or resistant) to either meropenem or imipenem according to Clinical and Laboratory Standards Institute (CLSI) 2017 breakpoints (Clinical and Laboratory Standards Institute, 2017). The two case groups were matched to a common control group of three controls, consisting of inpatients without HAIs, for (i) ward admitted to during hospital stay and (ii) time at risk (matched in blocks of five days; i.e. 1 – 5 days, 6 – 10 days, etc.). To ensure that the two parallel case-control models were comparable, an equal number of species type were selected for each group. Time at risk was defined as number of days of hospitalisation from admission to isolation of Enterobacterales of interest for case patients, and number of days from admission to discharge for controls.

### Data Collection

All data was sought from electronic medical records. Potential risk factors collected were: 1) patient demographics; 2) comorbidities (Charlsons co-morbidity index); 3) previous hospitalisations and length of stay; 4) previous admissions to intensive care unit (ICU) and ICU length of stay; 5) exposure to invasive devices (e.g. central venous catheter (CVCs), indwelling urinary catheters (IDCs), or drainage devices); 6) exposure to non-surgical invasive procedures; 7) exposure to surgical interventions; 8) exposure to surgical implants; 9) receipt of immunosuppressive therapy; and 10) receipt of antibiotics and duration (Charlson et al., 1994). Receipt of immunosuppressive therapy was defined as receipt of ≥1 dose of chemotherapy or immunosuppressants, or >14 days of corticosteroids at an equivalent daily dose of 20 mg prednisolone (Liu et al., 2013). Drainage devices include any drains used for wounds (traumatic or surgical) and in the peritoneal and pleural spaces for the purpose of removing bacteria and necrotic material. For prior antibiotic exposures, only antibiotics with activity against Enterobacterales were assessed. All risk factors were evaluated for an interval of 90 days prior to the occurrence of CnSE/CSE-HAI for case patients, or for an interval of 90 days prior to the date of discharge for controls.

### Microbiology

MICs of all tested antibiotics were determined for the CnSE isolates using commercial custom-made broth microdilution panels (Trek Diagnostics, East Grinstead, United Kingdom). Categorical susceptibility was determined according to the CLSI breakpoints (Clinical and Laboratory Standards Institute, 2017). Whole genome sequencing was employed to describe the sequence types (STs) and presence of carbapenemases for CnSE isolates. Briefly, genomic DNA were prepared from overnight bacterial cultures and extracted with the Qiagen Blood DNeasy kit (Hilden, Germany) before paired-end sequencing was conducted using Illumina systems (Illumina Inc., San Diego, CA). The reads were adaptors-removed and quality-trimmed using Trimmomatic software prior to *de novo* genome assembly using SPAdes (v.3.11.1). STs were determined by performing a basic local alignment search tool (BLAST) search of the assembled contigs against multilocus sequence typing (MLST) database (https://pubmlst.org/databases/). The SRST2 software tool were used to detect acquired antimicrobial resistance genes using ARGannot database (Inouye et al., 2014).

### Outcome Analysis

Clinical outcomes were collected for the case groups. The primary outcome was all-cause 30-day mortality. The secondary outcomes were all-cause in-hospital mortality, and time to discharge after infection for all patients and for patients discharged alive from the admission from interest. To adjust for factors that may confound the outcomes, details pertaining to severity of infection [as determined by the Sequential Organ Failure Assessment (SOFA) score], infection type, receipt of antibiotic with reported susceptibility, and presence of source control were collected. Source control was deemed to be appropriate when devices associated with the infection of interest were removed, or when interventions were made to remove the infected tissue (e.g. amputation). Patients were deemed to have received appropriate antibiotics for the infection if at least one antibiotic with reported susceptibility to the Enterobacterales based on the 2017 CLSI guidelines was prescribed for ≥48 hours (Clinical and Laboratory Standards Institute, 2017).

### Statistical Analysis

All statistical analyses were performed using Stata 14.0 (StataCorp, Texas, USA). Sample size calculations were informed by Teo et al. (2012) Using beta-lactam/beta-lactamase inhibitors as the exposure of interest, with an α level of 0.05, and a power of 0.80, we estimated that 74 patients in each case group will be needed to detect a moderate effect size (odds ratio of 3.0). Categorical variables were presented as numbers and percentages. Continuous variables were presented as median and interquartile range (IQR). In the risk factor analysis, univariate conditional logistic regression models were first used to compare each case group to control group. Odds ratios (OR) and 95% confidence intervals (CI) were calculated to evaluate the strength of association. Clinically plausible variables identified in the univariate analysis were included in a multivariable conditional logistic regression model in a step-wise selection manner if p ≤ 0.1. For risk factors where both presence and duration of exposure were collected (i.e. prior hospital or ICU stay, prior antibiotic use), the model was performed in two parts. First, presence of exposure to the risk factor of interest was compared for the case and control groups. Next, the duration of exposure was only compared if there is a positive binary relationship for exposure (i.e. the estimated marginal effects of the exposure on outcome had a p-value ≤0.1). Inclusion of risk factors into the final multivariable models was selected based on biologic plausibility and minimisation of the Akaike’s information criterion (AIC) (Vrieze, 2012). Results from the multivariable conditional logistic regression models for each case groups were compared to identify the risk factors independently associated with CnSE-HAIs. Model fit for the multivariable logistic regression models and the multivariable cox regression models were described using the Hosmer-Lemeshow goodness-of -fit test and the likelihood ratio test respectively. For outcomes analysis, mortality was analysed using logistic regression, while time to discharge was analysed using cox regression. Outcomes were adjusted for differences in severity of infection, infection type, antibiotic treatment and presence of source control. A final two-tailed p-value of <0.05 was considered statistically significant.

## Results

### Study Population

A total of 80 patients with CnSE-HAIs and 80 patients with CSE-HAIs were matched to 240 patients with no HAIs. Out of 80 Enterobacterales in CSE-HAI group, 56 (70.0%) were *Klebsiella* spp., 15 (18.8%) were *Enterobacter* spp., and 9 (11.3%) were *E. coli*. The demographics, comorbidities, prior healthcare and antibiotic exposure, and results of the univariate analysis are shown in **Table 1**. Compared to controls, significantly more CnSE-HAI patients had previous hospitalisation, ICU stay, prior surgical and instrumentation exposures, and received anti-pseudomonal beta-lactam/beta-lactamase inhibitors, 3^rd^/4^th^ generation cephalosporins, carbapenems, fluoroquinolones, and aminoglycosides.

**Table 1:** Univariate comparison of potential risk factors between (a) patients with CnSE- HAIs and patients with no HAIs, and (b) patients with CSE-HAIs and patients with no HAIs.

Characteristics	CnSE-HAI	CSE-HAI	No HAI	CnSE-HAI versus no HAI	CSE-HAI versus no HAI
No (%) or Median (IQR)	(n = 80)	(n = 80)	(n = 240)	OR (95% CI)	OR (95% CI)
Demographics and comorbidities					
Age, years	64 (57 – 72)	65 (57 – 71)	65 (54 – 76)	1.00 (0.98 – 1.01)	1.00 (0.99 – 1.02)
Male gender	45 (56.3)	50 (62.5)	140 (58.3)	0.92 (0.55 – 1.53)	1.18 (0.71 – 1.95)
Ethnicity					
Chinese	57 (71.3)	59 (73.8)	178 (74.2)	ref.	ref.
Malay	6 (7.5)	5 (6.3)	19 (7.9)	0.79 (0.33 – 1.89)	0.89 (0.38 – 2.08)
Indian	7 (8.8)	8 (10.0)	27 (11.3)	0.94 (0.35 – 2.51)	0.80 (0.28 – 2.24)
Others	10 (12.5)	8 (10.0)	16 (6.7)	1.96 (0.85 – 4.56)	1.49 (0.62 – 3.13)
Charlson comorbidity index	5 (3 – 6)	5 (3 – 7)	4 (2 – 7)	1.04 (0.95 – 1.14)	1.08 (0.99 – 1.19)
Time at risk	20 (10 – 39)	20 (9 – 38)	19 (9 – 35)	1.18 (0.47 – 2.93)	0.87 (0.37 – 2.04)
Prior hospital and instrumentation exposures					
Hospital stay	48 (60.0)	43 (53.8)	73 (30.4)	**3.22 (1.91 – 5.40)**	**2.67 (1.57 – 4.54)**
Duration of hospital stay, days	3 (0 – 14)	3 (0 – 14)	0 (0 – 4)	**1.03 (1.01 – 1.05)**	**1.04 (1.01 – 1.06)**
ICU stay	35 (43.8)	27 (33.8)	52 (21.7)	**5.64 (2.66 – 11.96)**	**2.60 (1.30 – 5.24)**
Duration of ICU stay, days	0 (0 – 8.5)	0 (0 – 3)	0 (0 – 0)	**1.22 (1.11 – 1.34)**	**1.09 (1.03 – 1.16)**
Surgery	61 (76.3)	53 (66.3)	118 (49.2)	**4.27 (2.22 – 8.23)**	**2.21 (1.25 – 3.91)**
Abdominal surgery	29 (36.3)	23 (28.8)	38 (15.8)	**3.73 (1.94 – 7.18)**	**2.44 (1.26 – 4.72)**
Surgery with implants	11 (13.8)	9 (11.3)	12 (5.0)	**3.35 (1.32 – 8.50)**	**3.09 (1.06 – 9.05)**
Endoscopy	38 (47.5)	29 (36.3)	49 (20.4)	**3.93 (2.18 – 7.07)**	**2.34 (1.31 – 4.20)**
Central line	55 (68.8)	38 (47.5)	68 (28.3)	**9.74 (4.67 – 20.27)**	**3.15 (1.66 – 5.95)**
Urinary catheter	36 (45.0)	28(35.0)	52 (21.7)	**2.99 (1.73 – 5.17)**	**2.13 (1.17 – 3.89)**
Drainage devices	44 (55.0)	27 (33.8)	51 (21.2)	**5.35 (2.91 – 9.86)**	**2.10 (1.14 – 3.87)**
Endotracheal intubation	31 (38.8)	25 (31.2)	42 (17.5)	**5.36 (2.52 – 11.42)**	**2.80 (1.42 – 5.44)**
Receipt of immunosuppressants	20 (25.0)	18 (22.5)	22 (9.2)	**4.68 (2.06 – 10.60)**	**4.04 (1.75 – 9.35)**
Presence of prior antibiotic use					
Penicillins	11 (13.8)	10 (12.5)	19 (7.9)	1.89 (0.84 – 4.24)	1.68 (0.74 – 3.84)
Anti-pseudomonal beta-lactam/beta-lactamase inhibitors	49 (61.3)	35 (43.8)	50 (20.8)	**11.61 (5.15 – 26.17)**	**3.27 (1.83 – 5.85)**
1^st^/2^nd^ generation cephalosporins	16 (20.0)	11 (13.8)	36 (15.0)	1.48 (0.74 – 2.96)	0.90 (0.43 – 1.89)
3^rd^/4^th^ generation cephalosporins	43 (53.8)	25 (31.3)	63 (26.3)	**3.38 (1.96 – 5.83)**	1.28 (0.73 – 2.25)
Carbapenems	44 (55.0)	24 (30.0)	21 (8.8)	**12.75 (6.80 – 23.89)**	**4.83 (2.38 – 9.79)**
Fluoroquinolones	31 (38.8)	21 (26.3)	43 (17.9)	**3.17 (1.74 – 5.77)**	1.73 (0.91 – 3.28)
Aminoglycosides	22 (27.5)	15 (18.8)	22 (9.2)	**4.44 (2.11 – 9.36)**	**2.29 (1.11 – 4.70)**
Duration of prior antibiotic exposure, days					
Anti-pseudomonal beta-lactam/beta-lactamase inhibitors	3 (0 – 11)	0 (0 – 6)	0 (0 – 0)	**1.13 (1.08 – 1.20)**	**1.08 (1.02 – 1.14)**
3^rd^/4^th^ generation cephalosporins	2 (0 – 7)	0 (0 – 4)	0 (0 – 1)	**1.05 (1.01 – 1.09)**	…
Carbapenems	3 (3 – 13)	0 (0 – 4)	0 (0 – 0)	**1.09 (1.05 – 1.14)**	**1.04 (1.01 – 1.08)**
Fluoroquinolones	0 (0 – 5)	0 (0 – 2)	0 (0 – 0)	**1.05 (1.01 – 1.09)**	…
Aminoglycosides	0 (0 – 1)	0 (0 – 0)	0 (0 – 0)	1.09 (0.99 – 1.19)	1.05 (0.95 – 1.16)

Factors with p-value ≤ 0.05 in bold.

CI, confidence interval; CnSE, carbapenem-nonsusceptible Enterobacterales; CSE, carbapenem-sensitive Enterobacterales; HAI, healthcare-associated infection; ICU, intensive care unit; IQR, interquartile range.

### Characteristics of CnSE-HAIs

The most common HAIs caused by CnSE were intra-abdominal infections (23/80, 28.8%), followed by respiratory infections (19/80, 23.8%) and skin and soft tissue infections (15/80, 18.8%). Out of the 80 patients with CnSE-HAIs, 23 (28.8%) were also identified as CRE carriers, by rectal swabs and/or stool samples. A total of 68 CnSE isolates (46 *Klebsiella* spp., 13 *Enterobacter* spp., and 9 *E. coli*) were available for phenotypic and genotypic characterisation (**Table 2**). Raw reads were deposited in the NCBI Sequence Read Archive (SRA) under study accession number PRJNA577535 and PRJNA751707. Most isolates were not susceptible to aztreonam, cefepime and piperacillin-tazobactam, but remained susceptible to amikacin, polymyxin B and tigecycline (**Table 2**). Polymyxin B MICs for the CnSE isolates ranged from ≤0.25mg/l – 16mg/l, while tigecycline MICs ranged from ≤0.25mg/l – 8mg/l. More than half of the CnSE isolates (45/68, 66.2%) were carbapenemase producers (**Table 2**). KPC-2 (21/45, 46.7%) was the most common carbapenemase; 1/45 (2.2%) harboured more than one carbapenemases. The MLST analyses of the 68 CnSEs showed diverse STs in all three species. CnS-*Klebsiella* spp. belonged to 29 different STs, with ST11 (n=7) and ST43 (n=4) being the most prevalent. CnS-*Enterobacter* spp. and CnS-*E. coli* belong to 10 and 8 different STs respectively, with ST93 (n=3) being the most prevalent for CnS-*Enterobacter* spp.

**Table 2:** Phenotypic and genotypic characteristics of the CnSE isolates.

Organism	*Klebsiella* spp. (n = 46)			*Escherichia coli* (n = 9)				*Enterobacter* spp. (n = 13)		
Genotypic characteristics										
Molecular mechanisms of resistance	*bla*_KPC-2_ (n = 11)				*bla*_KPC-2_ (n = 3)			*bla*_KPC-2_ (n = 7)		
	*bla*_OXA-48_ (n = 5)				*bla*_NDM-5_ (n = 2)			*bla*_NDM-1_ (n = 3)		
	*bla*_OXA-181_ (n = 2)				*bla*_OXA-181_ (n = 2)			*bla*_IMP-4_ (n = 2)		
	*bla*_OXA-232_ (n = 1)				non-CP CRE (n = 2)			CP co-producer (*bla*_NDM-7_ and *bla*_OXA-48_) (n = 1)		
	*bla*_NDM-1_ (n = 2)									
	*bla*_NDM-7_ (n = 1)									
	*bla*_IMP-4_ (n = 2)									
	*bla*_IMP-7_ (n = 1)									
	non-CP CRE (n = 21)									
Phenotypic characteristics										
Antibiotics	No. of NS isolates (%)	MIC_50_ (mg/L)	MIC_90_ (mg/L)		No. of NS isolates (%)	MIC_50_ (mg/L)	MIC_90_ (mg/L)	No. of NS isolates (%)	MIC_50_ (mg/L)	MIC_90_ (mg/L)
Amikacin	12 (26.1)	4	≥128		2 (22.2)	8	32	1 (7.7)	4	16
Aztreonam	44 (95.7)	≥64	≥64		9 (100)	≥64	≥64	13 (100)	≥64	≥64
Cefepime	39 (84.8)	≥64	≥64		9 (100)	≥64	≥64	13 (100)	≥64	≥64
Doripenem	40 (87.0)	8	≥32		7 (77.8)	8	≥32	11 (84.6)	4	≥32
Imipenem	40 (87.0)	8	≥32		7 (77.8)	16	≥32	11 (84.6)	8	≥32
Meropenem	41 (89.1)	8	≥32		7 (77.8)	≥32	≥32	11 (84.6)	8	≥32
Levofloxacin	28 (60.9)	8	≥32		8 (88.9)	16	≥32	3 (23.1)	1	≥32
Piperacillin-tazobactam	45 (97.8)	≥128	≥128		9 (100)	≥128	≥128	11 (84.6)	≥128	≥128
Polymyxin B	3 (6.5)	0.5	1		0 (0)	0.5	0.5	3 (23.1)	0.5	16
Tigecycline*^a^*	6 (13.0)	1	4		0 (0)	≥0.25	0.5	0 (0)	0.25	1

^a^Susceptibility defined as ≤2mg/L, according to the Food and Drug Administration breakpoints for Enterobacterales.

CP, carbapenemase producer, CnSE, carbapenem-nonsusceptible Enterobacterales, MIC_50_, lowest concentration at which 50% of the isolates were inhibited; MIC_90_, lowest concentration at which 90% of the isolates were inhibited.

### Risk Factor for CnSE-HAIs

The risk factors included into the multivariable models are presented in **Table 3**. Compared to controls, longer duration of prior ICU stay, prior use of surgical drains, prior exposure to anti-pseudomonal beta-lactam/beta-lactamase inhibitors, and prior exposure to carbapenems were independently associated with the development of CnSE-HAIs. Prior hospitalisation, longer duration of prior ICU stay, prior surgery, and prior exposure to anti-pseudomonal beta-lactam/beta-lactamase inhibitors were independently associated to the development of CSE-HAIs compared to controls. When comparing the two models, prior use of drainage devices and prior exposure to carbapenems emerged as unique risk factors for CnSE-HAIs.

**Table 3:** Multivariable comparison of potential risk factors between (a) patients with CnSE and patients with no HAIs, and (b) patients with CSE and patients with no HAIs.

Variables	CnSE versus no HAIs	CSE versus no HAIs
	aOR (95% CI)	aOR (95% CI)
Hospital stay	2.32 (0.96 – 5.63)	**3.02 (1.57 – 5.83)**
Duration of ICU stay	**1.25 (1.05 – 1.47)**	**1.10 (1.03 – 1.18)**
Surgery	2.29 (0.69 – 7.64)	**2.51 (1.25 – 5.03)**
Central line	…	1.48 (0.67 – 3.24)
Drainage devices	**2.19 (1.29 – 3.70)**	1.35 (0.95 – 1.92)
Exposure to anti-pseudomonal beta-lactam/beta-lactamases	**5.92 (1.87 – 18.7)**	**2.27 (1.15 – 4.48)**
Exposure to 3^rd^/4^th^ generation cephalosporins	2.29 (0.85 – 6.15)	…
Exposure to carbapenems	**17.09 (3.06 – 95.4)**	2.25 (0.97 – 5.24)

Factors with p-value ≤0.05 in bold.

Factors with p-value ≤0.05 in bold.

a Goodness of fit test: (χ^2^ = 144.7, d.f. = 7, p < 0.001).

b Goodness of fit test: (χ^2^ = 63.1, d.f. = 7, p < 0.001).

CI, confidence interval; CnSE, carbapenem-nonsusceptible Enterobacterales; CSE, carbapenem-sensitive Enterobacterales; HAI, healthcare-associated infection; ICU, intensive care unit.

### Outcomes of Patients With CnSE-HAIs

We compared the infection characteristics, treatment characteristics, and clinical and microbiological outcomes of CnSE-HAI and CSE-HAI patients (**Table 4**). Twenty-seven (33.8%) and 31 (38.8%) of CnSE-HAI and CSE-HAI patients respectively had bacteraemia. No significant differences were observed in the infection severity and types of infection between both case groups. Significantly less CnSE-HAI patients received antibiotics with reported susceptibility compared to CSE-HAI patients (90.0% vs. 98.8%). Forty-four (55.0%) of CnSE-HAI patients and 78 (97.5%) of CSE-HAI patients received monotherapy while 28 (35.0%) of CnSE-HAI patients and 1 (1.3%) of CSE-HAI patients had combination therapy. Twenty-six (32.5%) of CnSE-HAI patients and 1 (1.3%) of CSE-HAI patients had polymyxin-based therapy. The median time to receipt of antibiotics with reported susceptibility for CnSE-HAI and CSE-HAI patients were 3 (IQR: 1–4) and 2 (IQR: 1–3) days respectively.

**Table 4:** Infection characteristics, treatment characteristics, and outcomes of patients with (a) CnSE HAIs and (b) CSE HAIs.

Characteristics	CnSE HAIs	CSE HAIs	Unadjusted OR (95% CI)	Adjusted OR (95% CI)*^a^*
No (%) or Median (IQR)	(n = 80)	(n = 80)		
Infection characteristics				
SOFA score at infection onset	6 (4 – 9)	5 (4 – 8)	1.44 (0.44 – 4.38)	
Infection type				
Intra-abdominal	23 (28.8)	14 (17.5)	ref.	
Respiratory tract	19 (23.8)	18 (22.5)	0.64 (0.35 – 1.62)	
Skin and soft tissue	15 (18.8)	17 (21.3)	0.54 (0.21 – 1.40)	
Urinary tract	14 (17.5)	18 (22.5)	0.47 (0.18 – 1.24)	
Line	6 (7.5)	5 (6.3)	0.73 (0.19 – 2.85)	
Primary source unknown	3 (3.8)	8 (10.0)	0.23 (0.05 – 1.00)	
Presence of bloodstream involvement	27 (33.8)	31 (38.8)	0.81 (0.42 – 1.54)	
Treatment characteristics				
Receipt of antibiotics with reported susceptibility	72 (90.0)	79 (98.8)	0.11 (0.01 – 0.93)	0.06 (0.01 – 0.51)
Achieved appropriate source control	33 (41.3)	32 (40.0)	1.05 (0.56 – 1.98)	
Clinical outcomes of infection				
All-cause 30-day mortality	21 (26.3)	16 (20.0)	1.43 (0.68 – 2.99)	0.92 (0.37 – 2.28)*^d^*
All-cause in-hospital mortality	32 (40.0)	19 (23.8)	2.14 (1.08 – 4.23)	2.00 (0.82 – 4.86)*^e^*
Time to discharge after infection (all patients)*^b^*	23 (9 – 43)	21 (10 – 38)	0.80 (0.58 – 1.09)	0.71 (0.51 – 0.98)*^f^*
Time to discharge after infection among patients discharged alive*^c^*	29 (15 – 47)	21 (10 – 39)	0.69 (0.46 – 1.02)	0.66 (0.44 – 0.98)*^g^*

^a^Adjusted for differences in severity of infection, infection type, receipt of antibiotics with reported susceptibility and presence of source control between CnSE-HAI and CSE-HAI patients

^b^Includes patients who died during current admission and those discharged alive (n = 160).

^c^Includes only patients who were discharged alive from current admission (n = 109).

^d^Goodness of fit test: (χ^2^ = 48.2, d.f. = 4, p <0.001).

^e^Goodness of fit test: (χ^2^ = 29.7, d.f. = 4, p <0.001).

^f^Goodness of fit test: (χ^2^ = 15.7, d.f. = 4, p =0.003); Schoenfeld test for proportionality: (p = 0.59).

^g^Goodness of fit test: (χ^2^ = 9.3, d.f. = 4, p = 0.05); Schoenfeld test for proportionality: p = 0.92).

Factors with p-value ≤0.05 in bold.

CnSE, carbapenem-nonsusceptible Enterobacterales; CSE, carbapenem-sensitive Enterobacterales; HAI, healthcare-associated infection; ref, reference.

We did not observe significant differences in all-cause 30-day mortality (26.3% vs. 20.0%). All-cause in-hospital mortality rates in CnSE-HAI patients were significantly higher in the crude comparison (40.0% vs. 23.8%); however, this difference was not observed after accounting for differences in the receipt of antimicrobials with reported susceptibility (**Table 4**). CnSE-HAI patients had a significantly longer time to discharge compared to CSE-HAI patients, when all patients and patients discharged alive were compared; these differences remained after accounting for disease severity, receipt of antimicrobials with reported susceptibility and receipt of appropriate source control.

## Discussion

This study investigated the predictors and outcomes of CnSE-HAIs in Singapore. We found that use of drainage devices and carbapenem exposure were associated with CnSE-HAIs in Singapore hospitals. Compared to patients with CSE-HAIs, patients with CnSE-HAIs had a higher crude rate of in-hospital all-cause mortality and longer length of stay. The observed higher in-hospital all-cause mortality rates appeared to be independently associated to the lower rates of receipt of antibiotics with reported susceptibility in CnSE-HAI patients.

Studies on CRE risk factors, even when done in the same setting, can be challenging to compare. This is because subtle differences in design and definitions can cause findings to vary (von Elm et al., 2014). In local risk factor study conducted in 2015, the authors identified prior exposure to penicillins, glycopeptides, and presence of central lines as risk factors for CRE acquisition (Ling et al., 2015). Unlike the study, we did not evaluate antibiotics without activity against Enterobacterales as potential risk factors. This is because we think that these antibiotics are commonly prescribed for empiric broad-spectrum coverage alongside antibiotics with Enterobacterales activity. Any association of these antibiotics with CRE-HAIs could have been a coincidental finding.

A key finding in our study is any prior receipt of carbapenems (as opposed to duration of carbapenem use) is significant risk factor for development of CnSE-HAIs. This is most likely associated with the selective pressure on the microbial environment upon carbapenem exposure. Carbapenems kills beneficial flora and susceptible bacteria while bacteria resistant to carbapenems survive, multiply and become predominant, predisposing the host to a greater risk of infection by carbapenem-resistant organisms (Patel et al., 2011). Currently, all public hospitals in Singapore (and some private hospitals in Singapore) already have ASPs that utilise a multi-pronged approach to promote appropriate carbapenem use. Appropriate carbapenem use is largely promoted in these ASPs by reducing the duration of existing carbapenem prescriptions (e.g. concurrent audit and feedback) only after exposure to carbapenems has occurred (e.g. de-escalating to a narrower-spectrum antibiotic or restricting the duration of use). Our findings suggested that strategies to prevent unnecessary carbapenem exposure such as formulary restrictions and computerised decision support systems may play a more vital role in the prevention of CnSE-HAIs, compared to strategies that reduces the duration of carbapenem prescriptions after exposure has occurred (Barlam et al., 2016).

Prior use of drainage devices was another significant risk factor associated with CnSE-HAIs in our study. We postulate two possible explanations for this finding. Firstly, the drainage device could have acted as a portal of entry or source of infection, either from endogenous organisms present on the skin or exogenous bacteria from the environment. However, we believe that infections from the exogenous environment is less likely as most of the CnSE strains in our study were non-clonal, reducing the likelihood that development of CnSE-HAIs due to horizontal transmission from the environment. Endogenous infections from organisms present on the skin is also unlikely given that none of the other instrumentation/devices in our study were associated with increased risk of CnSE-HAIs. A second and more likely explanation is that the presence of drainage devices represents the presence of high microbial burdens at collection sites with limited antimicrobial penetration. This could have resulted in failure to eradicate the infection, leading to the selection of the resistant subpopulations present within the microbial collection (Harada et al., 2014).

We observed that the significantly higher mortality in CnSE-HAI patients, which was independently associated with the lower rates of receipt of antimicrobial therapy with reported susceptibility. Our findings were similar to that of Lodise et al, who found in a systematic review that regardless of CRE status, patients who received delayed appropriate therapy had a greater likelihood of mortality. The reasons for lack of receipt of antibiotics with reported susceptibility in CnSE-HAI patients were likely to be multifactorial. Firstly, patients with CSE-HAIs would have likely received empiric antibiotics with adequate coverage, but the same empiric antibiotics would have unlikely been able to adequately target HAIs caused by CnSE. Hence, CnSE-HAI patients that were critically ill may have died before the availability of comprehensive antimicrobial susceptibility data and receipt of antibiotics with reported susceptibility. Secondly, even after the CnSE status is known, there may have been a lack of antibiotic agents with adequate *in vitro* activity against the CnSE-HAI, especially in patients where existing comorbidities may have precluded the limited remaining effective antibiotic armamentarium (e.g. tigecycline cannot be prescribed to patients with severe hepatic dysfunction). Our results suggest that there is a need to evaluate the utility of new or resurrected agents such as ceftazidime (or aztreonam)/avibactam, imipenem/relebactam, meropenem/vaborbactam, plazomicin and intravenous fosfomycin as potential empiric therapy in patients with high risk of CnSE-HAIs in our local setting (Michalopoulos et al., 2011; Shields et al., 2017). Efforts should also be directed at improving antimicrobial therapy through use of rapid resistance diagnostics and clinical prediction tools to identify patients with greatest risk for CnSE-HAIs (Burnham et al., 2017).

As with many antimicrobial resistance epidemiological studies, this study has limitations. Firstly, the retrospective nature of our study meant that the risk factors collected was contingent on the completeness of prior recordkeeping. For instance, while we could accurately determine the presence/absence of instrumentations, we were unable to further explore the duration of the instrumentations as such data was inconsistently available. Secondly, we were unable to collect risk factors associated with potential environmental spread and acquisition of CnSE (e.g. contact with healthcare personnel or patients who are CRE colonisers). However, our results here indicate a diversity of sequence types and carbapenemases, suggesting that CnSEs observed in our study is less likely due to clonal expansion. In our MSLT analysis, we were unable to conduct in-depth plasmid analyses to detect carbapenemase dissemination *via* plasmid spread due to the limitations of short-read sequencing. Further investigations utilizing long-read sequencing will be valuable in elucidating the transmission mechanisms of carbapenemases among CREs in Singapore hospitals. Thirdly, we did not collect the susceptibilities data of non-carbapenem antibiotics of the bacterial pathogens from both CSE-HAI and no HAI groups, and the identities of causative pathogens of community acquired infections in no HAI group.

In conclusion, nosocomial infections caused by CRE constitute a significant clinical and public health threat due to their propensity for spread and limited treatment options (Nordmann et al., 2012). Our study findings suggested that appropriate management of deep-seated Enterobacterales infections, and enhancement of antimicrobial stewardship strategies to prevent carbapenem exposure may be useful in reducing the risk of CnSE-HAIs. Efforts should also be made to improve antimicrobial therapy in patients, possibly through use of rapid resistance diagnostics and clinical prediction tools to identify patients with the greatest risk for CnSE-HAIs, to improve outcomes of patients with CnSE-HAIs.

## Data Availability Statement

The sequencing data has been deposited in the NCBI Sequence Read Archive (SRA) under study accession numbers PRJNA577535 and PRJNA751707.

## Ethics Statement

The studies involving human participants were reviewed and approved by SingHealth and National Healthcare Group ethics review board (CIRB/2016/2388). Written informed consent for participation was not required for this study in accordance with the national legislation and the institutional requirements.

## Author Contributions

GH: Methodology, Investigation, Data curation, Writing – original draft. YC: Conceptualisation, Methodology, Formal analysis, Writing – review and editing, Project administration. YQ: Investigation, Data curation. JT: Resources, Writing – review and editing. SC: Resources, Writing – review and editing. TK: Resources Writing – review and editing. TL: Resources, Writing – review and editing. KM: Resources, Writing – review and editing. ON: Resources, Writing – review and editing. AK: Conceptualisation, Resources, Writing – review and editing, Supervision, Funding acquisition. All authors contributed to the article and approved the submitted version.

## Funding

This study was supported by the National Medical Research Council Centre Grant (NMRC/CG/M011/2017), the National Medical Research Council Centre Grant (NMRC/CG/C005B/2017), and the Singapore General Hospital Research Grant (SRG-AN#01/2016).

## Conflict of Interest

The authors declare that the research was conducted in the absence of any commercial or financial relationships that could be construed as a potential conflict of interest.

## Publisher’s Note

All claims expressed in this article are solely those of the authors and do not necessarily represent those of their affiliated organizations, or those of the publisher, the editors and the reviewers. Any product that may be evaluated in this article, or claim that may be made by its manufacturer, is not guaranteed or endorsed by the publisher.
